# Viper’s bugloss (*Echium* spp.) honey typing and establishing the pollen threshold for monofloral honey

**DOI:** 10.1371/journal.pone.0185405

**Published:** 2017-10-04

**Authors:** Tomás Martín Arroyo, Amelia V. González-Porto, Carmen Bartolomé Esteban

**Affiliations:** 1 Laboratorio de Miel y Otros Productos de la Colmena, Centro de Investigación Apícola y Agroambiental de Marchamalo, Instituto Regional de Investigación y Desarrollo Agroalimentario y Forestal, Junta de Comunidades de Castilla La Mancha, Guadalajara, Spain; 2 Departamento de Ciencias de la Vida (Botánica), Facultad de Biología, C. Ambientales y Químicas, Universidad de Alcalá, Madrid, Spain; University of Cologne, GERMANY

## Abstract

Honey samples (n = 126) from Castilla-La Mancha (Central Spain) were characterized based on their physicochemical properties and a melissopalynological analysis. The latter showed that *Echium* pollen type was the dominant palynomorph in most samples, representing at least 30% of the pollen in each sample. As anticipated, a relationship was observed between the proportion of this pollen and the properties of the honey. One goal of this study was to set a threshold that defines the percentage of pollen necessary for Viper’s bugloss honey to be considered monofloral or multifloral. This is a mandatory requirement in light of the publication of the European Directive 2014/63/EU establishing the regulations governing the labelling and control of honey to eradicate fraud (BOE n° 147, June 2015). By analyzing how the proportions of *Echium* pollen type affected the physicochemical and sensory parameters of the honey, the honeys analyzed could be segregated into multifloral and monofloral honeys. The data indicates that the proportion of pollen necessary to discriminate monofloral Viper’s bugloss honey lies at 70%.

## Introduction

While the Borage family is native to southern Europe, the members of this family also occur in most countries worldwide. The species of the family Boraginaceae are clearly entomophilous, attracting insects with the reward of both pollen and nectar. The position of their anthers and the smallness of the grains ensures that large amounts of pollen are also released into the atmosphere, with its consequent anemophilous transport. In fact, these plants may free about 35.9 grams of pollen grains per cubic meter at atmosphere, although this varies depending on climatology [1].

The *Echium* spp. are wild plants that prosper in dry meadows and fields, waste ground and roadsides. They are an important nitrophile in pastures and meadows across the Iberian Peninsula [2] and their wildflowers are blue, or occasionally white or pink, appearing from late spring to mid-summer. According to Flora Ibérica [3], seven species of the genus *Echium* can be found in Castilla-La Mancha (Central Spain): *E*. *boissieri* Steudel, *E*. *creticum* L., *E*. *flavum* Desf., *E*. *asperrimum* Lam; *E*. *humile* Desf.; *E*. *vulgare* L., *E*. *creticum* L. and *E*. *plantagineum* L. Of these, the most abundant is without doubt *E*. *plantagineum* L., followed by *Echium vulgare*.

Palynologically, most of these species produce the same pollen type described by Díez [4,5], characteristically small in size (P = 13–25μm; E = 8–15μm) and heteropolar. While this facilitates their rapid identification, it does not favor palynological identification of the different species in honey. *Echium* species produce large amounts of pollen. In the case of *E*. *albicans*, the density of flowers reaches 14,639 (±3,720)/m^2^ with a production of 123,331 (±32.12) pollen grains per flower, whereas the density of flowers in the case of *E*. *plantagineum* reaches 27,845 ± 4,927/m^2^ and the pollen grains 97,571 ± 21,456 [6,7]. If we compare these numbers of pollen grains per flower with those of the Lamiaceae (Labiatae) family, such as *Rosmarinus officcinalis* L. or *Thymus*, the contribution of the pollen of these latter apiculture sources (nectariferous plants) is much lower: *R*. *officcinalis*, pollen contributes 8,300 grains; *T*. *mastichyna* (L.) L. 4,321; and *Teucrium fruticans* L., 13,999.

The *Echium* spp. are assiduously sought out by bees and bumblebees also due to the large amount of nectar they produce [8,9]. However, little is known about the composition of the nectar in Mediterranean communities and plants. Several aspects of pollination are influenced by climate, such as nectar quantity and concentration, flowering time and pollinator assemblages [10]. Indeed, nectary morphology, as well as the amount and concentration of nectar sugars, are related to the type of pollinator a plant is visited by [11–13]. In the case of *E*. *plantagineum*, each flower receives around 5,955 visits throughout the day, 90.2% of the time from the Apidae family. Of these, 87.8% correspond to *Bombus terrestres*, which visits 23.1 flowers per minute, and 2.4% to *Apis mellifera*, visiting approximately 15.4 flowers per minute [14].

In this study we set out to define different parameters of *Echium* (Viper′s bugloss) flower honey. Honey is a viscous and appreciated aromatic product prepared by bees mainly from flower nectar or honeydew [15]. In ancient Greece, it was believed that honey promoted longevity and virility, and there are written reports of its use in traditional medicine in ancient Egypt (5,000 years ago), as well as in ancient China and even Russia. The characteristics of honey, including its appearance, flavor, sweetness and texture, as well as its medicinal properties, attract thousands of consumers [15]. Although there is considerable variation in honey composition depending on the edaphic and botanical origin [16], the typical composition of honey is: moisture, 20.0%; carbohydrates, 79.7%; proteins, 0.2%; and ash, 0.1% [17]. Honey also contains a number of components that act as conservatives, such as vitamin C, flavonoids and other phenols, as well as enzymes like glucose oxidase, catalase and peroxidase [18]. Indeed, it has been suggested that the conservative properties of some of these substances is due to their antioxidant activity [19,20].

Honey has been termed a value added product ever since the initial studies confirmed that the antioxidant properties of polyphenols lie at the heart of their cosmetic [21–23], medical [24–26] and alimentary applications [27]. Indeed, antioxidant supplementation is beneficial in preventing certain diseases. Antioxidant capacity is generally assessed by two main methods, the quenching of various free radicals or the reduction of metal ions like iron, copper and chromium [28]. Hence, it is clearly important to determine the specific properties of each type of honey [17].

Significantly, *E*. *vulgare* is also a traditional medicinal herb that is used to treat kidney and respiratory diseases, to soothe irritated tissues, and to aid in wound healing. In Spain, this plant has been used in traditional medicine as an anti-catarrhal agent [29] and there are references to the properties of *E*. *plantagineum* to treat stomach ache [30]. Moreover, it is often one of the many blossoms contributing to multifloral honeys worldwide, accounting for at least 45% of the content except in monofloral honeys [31]. Accordingly, it is important to determine the precise proportions of pollen necessary to discriminate *Echium* monofloral honeys, and to quantify and specify the characteristics of viper’s bugloss honey in the Iberian Peninsula. Defining the features necessary to classify viper’s bugloss honey as monofloral will aid its labeling, thereby helping to control fraud [32], and it will help consumers who are looking for specific properties in the honey they consume, in line with the European Directive 2014/63/UE.

## Materials and methods

### Location of beehives and definition of the study area

**Fig 1:**
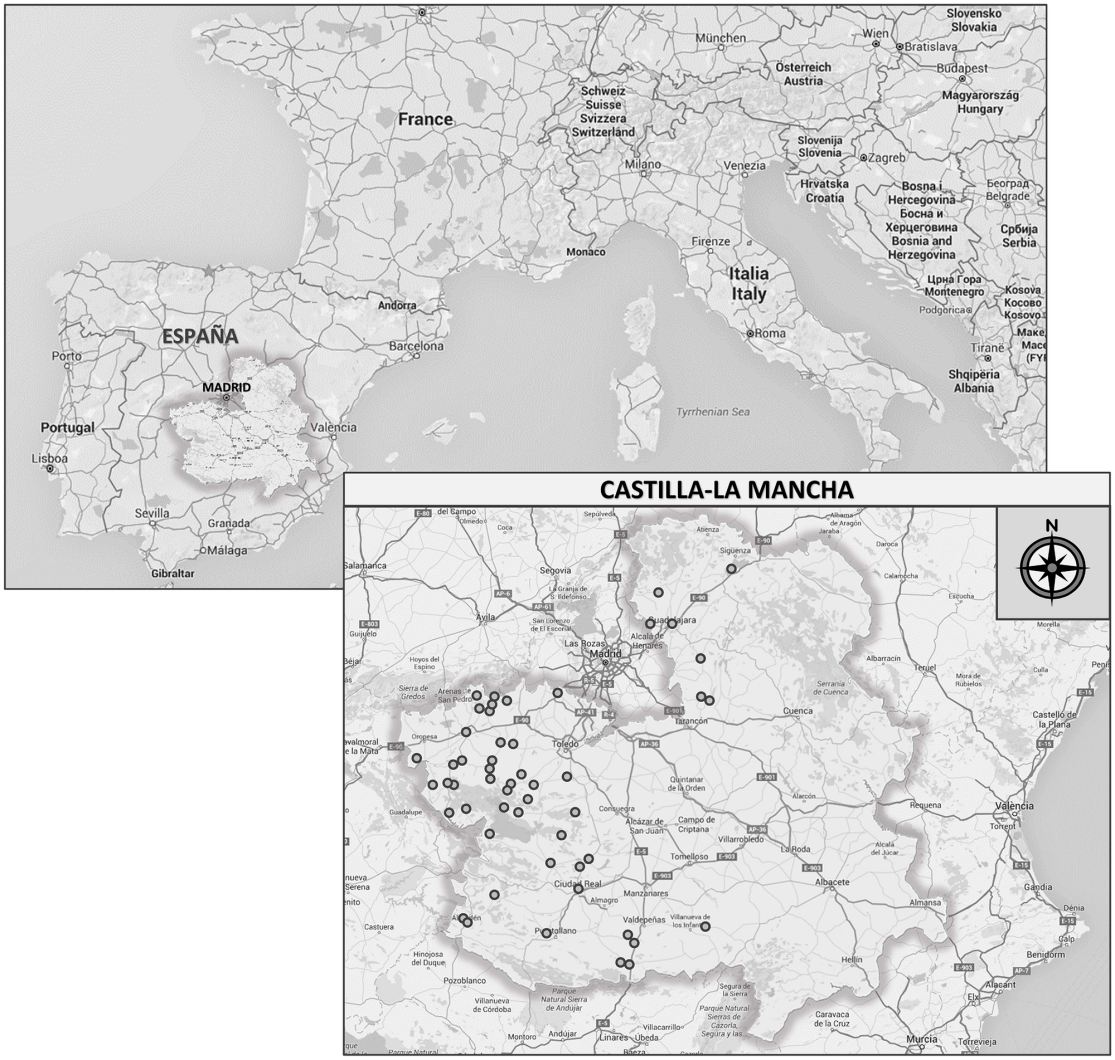
Map of “Castilla La Mancha” showing the sampling sites.

The honey samples were collected directly by beekeepers in different areas of Castilla-La Mancha, Spain (Fig 1, Table 1). There is no transhumance of the hives in this region and the honey is extracted by centrifugation of the combs. The beehives are located in open areas dominated by herbaceous (annual and biannual) and woody communities, indicating impaired land use. Hence, perennial plants that contribute to shrub substitution (*Thymus*, *Lavandula*, etc.) are represented in the honey, as well as other plants (*Echium plantagineum*, *E*. *vulgare*, *Brassica*, *Diplotaxis*, etc.), which indicate soil subnitrification or nitrification. Nevertheless, the most abundant plant is without any doubt *E*. *plantagineum*, a major component of the nitrophiles.

**Table 1:** Production sites and geographical coordinates.

PRODUCTION SITES		LATITUDE	LONGITUDE
Almaden	Ciudad Real	38.774464	-4.832621
Almodovar Del Campo	Ciudad Real	38.70828	-4.175695
Almuradiel	Ciudad Real	38.513078	-3.496555
Anchuras	Ciudad Real	39.479808	-4.837157
Chillon	Ciudad Real	38.823685	-4.872809
Cortijos (Los)	Ciudad Real	39.314464	-4.051939
Encomienda De Mudela	Ciudad Real	38.641947	-3.464775
Fernan Caballero	Ciudad Real	39.123329	-3.901818
Granatula De Calatraba	Ciudad Real	38.798713	-3.742239
Horcajo De Los Montes	Ciudad Real	39.327835	-4.64891
Malagon	Ciudad Real	39.166806	-3.853225
Porzuna	Ciudad Real	39.146809	-4.155215
Retuerta Del Bullaque	Ciudad Real	39.463764	-4.411779
Saceruela	Ciudad Real	38.945204	-4.607595
Santa Cruz De Mudela	Ciudad Real	38.641078	-3.467508
Villahermosa	Ciudad Real	38.750094	-2.871427
Viso Del Marques	Ciudad Real	38.521597	-3.562291
Saceda-Trasierra	Cuenca	40.154047	-2.853658
Algora	Guadalajara	40.961388	-2.666711
Cantalojas	Guadalajara	41.235902	-3.245163
Illana	Guadalajara	40.183198	-2.908477
Malaguilla	Guadalajara	40.821234	-3.258991
Pastrana	Guadalajara	40.416124	-2.921341
Valdeavero	Guadalajara	40.633257	-3.33287
Alcaudete De La Jara	Toledo	39.785595	-4.868919
Aldeanueva De San Bartolome	Toledo	39.636348	-5.110638
Almendral De La Cañada	Toledo	40.183349	-4.740042
Belvis De La Jara	Toledo	39.760413	-4.948077
Buenasbodas	Toledo	39.642705	-4.948727
El Carpio De Tajo	Toledo	39.888132	-4.456805
Garciotur	Toledo	40.098992	-4.646972
Hinojosa De San Vicente	Toledo	40.104194	-4.723873
Hontanar	Toledo	39.612367	-4.498327
La Nava De Ricomalillo	Toledo	39.63785	-4.987049
Los Navalmorales	Toledo	39.725507	-4.641161
Los Navalucillos	Toledo	39.664984	-4.642452
Malpica De Tajo	Toledo	39.897551	-4.548012
Mazarambroz	Toledo	39.694596	-4.019743
Menasalbas	Toledo	39.641411	-4.283342
Montes De Toledo	Toledo	39.55	-4.333333
Navahermosa	Toledo	39.635595	-4.472251
Navas De Estena	Toledo	39.493866	-4.519261
Nombela	Toledo	40.156354	-4.504445
Nuño Gomez	Toledo	40.114132	-4.619857
Pelahustan	Toledo	40.176256	-4.599207
San Martin De Montalban	Toledo	39.701862	-4.388637
San Martin De Pusa	Toledo	39.782966	-4.632208
San Pablo De Los Montes	Toledo	39.541629	-4.332111
Sevilleja De La Jara	Toledo	39.460882	-4.979902
Talavera De La Reina	Toledo	39.962884	-4.830454
Valdeverdeja	Toledo	39.797424	-5.246246
Valmojado	Toledo	40.204946	-4.088713

The sampling territory is located in two biogeographic provinces: the Western Mediterranean Iberian province that includes Northwest Toledo and Guadalajara, where *Quercus*. *suber* L., *Q*. *robur* L. and *Castanea sativa* Mill. are found; and the Central Mediterranean Iberian Province that includes Albacete, Ciudad Real, Cuenca and Guadalajara. In both these territories, *Quercus rotundifolia* Lam. oak woods dominate the vegetation.

### Samples

Samples of honey harvested consecutively from 2005 to 2013 were analyzed. The *Echium* honey was classified by melissopalynology and its sensorial characteristics were defined. Each of the honey samples (1 kg) was separated into two parts: one was stored at room temperature (18–22°C) and it was used for physicochemical and sensorial analyses; and the other was frozen at –30°C for further analysis. Except for the sensorial analyses, all the other characteristics were measured in duplicate. Of the 210 samples, 126 were selected in which *Echium* was present (157 samples in which *Echium* pollen represented at least 30% and 126 with more than 45% *Echium* pollen). At present, viper’s bugloss honey is considered monofloral when *Echium* pollen represents at least 45% of total pollen load [31].

### Pollen analysis

The pollen was analyzed based on previously described methods [31]. The honey samples were treated chemically with acidified water (sulfuric acid, 10%), and a qualitative and quantitative analysis was performed (Von der Ohe et al. [32]) on the sediment recovered from 5 g aliquots. A minimum of 300 pollen grains were counted in each sample (between 300 at 1200 pollen grains), and the pollen grains from each sample were identified and classified on the basis of the identification keys available at the Centro Agrario de Marchamalo (CAR) honey laboratory [33–37]. This resource was supplemented by the manual and digital pollen collection that contains pollen slides already available in the laboratory, as well as by new pollen slides produced from the material collected during this study. The International Commission for Plant-Pollinator Relationships’ (ICPPR) recommendations were followed to classify the honey according to its floral origin [31], bearing in mind the minimum percentages of nectariferous pollen for monofloral honeys.

A quantitative analysis was performed on an aliquot (10μL) of the sediment obtained, expressing the results as the number of pollen grains per gram of honey. The number of pollen grains in the aliquot was counted under light microscopy and the magnification that is most suitable for identifying the various elements in the sediment (400 to 1000 ×). A qualitative analysis was also performed on the same sediment. The results were expressed as percentages and divided into the following frequency classes: P, pollen present, always below 1% of the pollen spectrum; R, minor pollen, representing between 1% and 3%; I, important pollen, representing between 3% and 15%; A, accompanying or secondary pollen, representing between 15% and 45%; and D, dominant pollen, equivalent to or above 45%.

### Sensorial analysis

The sensory attributes were evaluated by a panel of trained honey analysts at the CAR. The 7 assessors on the sensory panel are trained according to the general guidelines (ISO 8586–1, [38], ISO 8586–2 [39] based on ISO 5492 [40], and they followed the protocol closest to that established and published in Apidologie by the research group for the sensorial description of monofloral honeys of the International Honey Commission (IHC: [33]). These analysts have 10 years of experience in describing honeys from the Alcarria and Castilla-La Mancha regions. The 7 assessors evaluated the color, odor (family odor and other olfactory perceptions, intensity and persistence), aftertaste, taste (salty, sweetness, bitterness and acidity), aroma, intensity and persistence and astringency of the honey, as well as the presence of crystallization. A descriptive score sheet was designed with a structured scale and a blank space where the assessors indicated their perceptions. The scale was designed with numerical intervals of 0–3 points and the assessors described the characteristics of each perception (especially family odor, secondary olfactory perceptions and taste attributes).

### Physicochemical analysis

The parameters for honey quality were determined using the methods adopted and reviewed by the IHC [41] Moisture was determined by refractometry using a refractometer (Abbe 325; Auxilab S.L., Navarra, Spain), and the color of the honey was measured spectrophotometrically and using a Lovibond apparatus/tintometer (expressed as Pfund values). The pH of a 10% (w/v) solution of honey prepared in freshly boiled distilled water was measured potentiometrically at 20°C using an Eutech System XS PC510 pH-meter. The free acidity was obtained by plotting the neutralization curve with a titrated NaOH solution and determining the pH of the equivalence point. Electrical conductivity was measured at 20°C on a 20% (w/v) solution of honey (dry matter basis) prepared in deionized water using a Radiometer CDM-83 conductimeter and the results were expressed as mScm^-1^. Hydroxymethylfurfural (HMF) content was determined using the White spectrophotometric method.

### Determination of the antioxidant capacity

The antioxidant activity was evaluated spectrophotometrically using the stable free radical DPPH test (1,1-diphenyl-2-picrylhydrazyl: see Vela et al., [42]).

### Vitamin C

The vitamin C content was determined using the 2,6-dichloroindophenol titrimetric method (AOAC method for juices). The AOAC method involves a redox titration with 2,6-dichloroindophenol [43]. The vitamin C content was determined by RP-HPLC in isocratic mode, with a mobile phase of 0.01% (v/v) H_2_SO_4_ (Panreac) at pH 2.5, a flow rate of 0.9 mL/min, and UV detection at 245 nm and 25 (± 1)°C (the method used in this work was adapted from Vázquez-Oderiz et al., [44].and León-Ruiz et al., [45]).

### Sugar profile

This approach aimed to identify parameters that discriminate between the different honey samples using a method described previously [45].The sugar content (glucose and fructose) was determined by HPLC but using refractometry detection (Merck RI-71). A mixture of acetonitrile:water (87:13% v/v) was used as the mobile phase at a flow rate of 1 mL/min. Separation was carried out at 40°C using a Lichrospher 100 NH2 (5 *μ*m: Merck Darmstadt, Germany). Honey samples were dissolved to 5% (w/v) in water and filtered through a nylon syringe filter (0.45 *μ*m).

### Statistical analysis

Of the 210 samples collected in the sampling area containing viper’s bugloss pollen, 157 had at least 30% representation and these were the samples used in the present study. Among these samples, there are data on 23 chemical and 40 pollen variables that define the general spectrum of viper’s bugloss honey, although only some define the specific character of these honeys. After applying a second filter that removes those samples that do not have data for at least 10% of the variables, the study population was reduced to 126 samples.

To discern which variables most strongly influence the monofloral character of this type of honey, an initial correlation analysis was carried out between the percentages of *Echium* pollen (Echium type will appoint as from this moment) and each of the variables. This initial approach identified the variables most strongly related to a high representation of *Echium* type pollen, allowing the most robust parameters that present the strongest relationships to be selected, whether directly or inversely proportional. Subsequently, groups and cohorts were established with variations in the presence of pollen from the species under analysis within the ranges of 3%, 5% and 10%. A Principal Components Analysis (PCA) and a Cluster analysis of these cohorts was used to establish, with the greatest possible precision, at what percentage of pollen there is a tighter relationship between the proportion of *Echium* and the physicochemical characteristics studied. The Variables diagram revealed the evolution of these characteristics with the change in the percentage of *Echium* pollen. These statistical and graphical analyses were carried out with specific software, including Biplot 1.1 [46] and Olea-DP [47] working on Microsoft Excel and Tilia [48,49] or Conniss [50].

## Results and discussion

**Fig 2:**
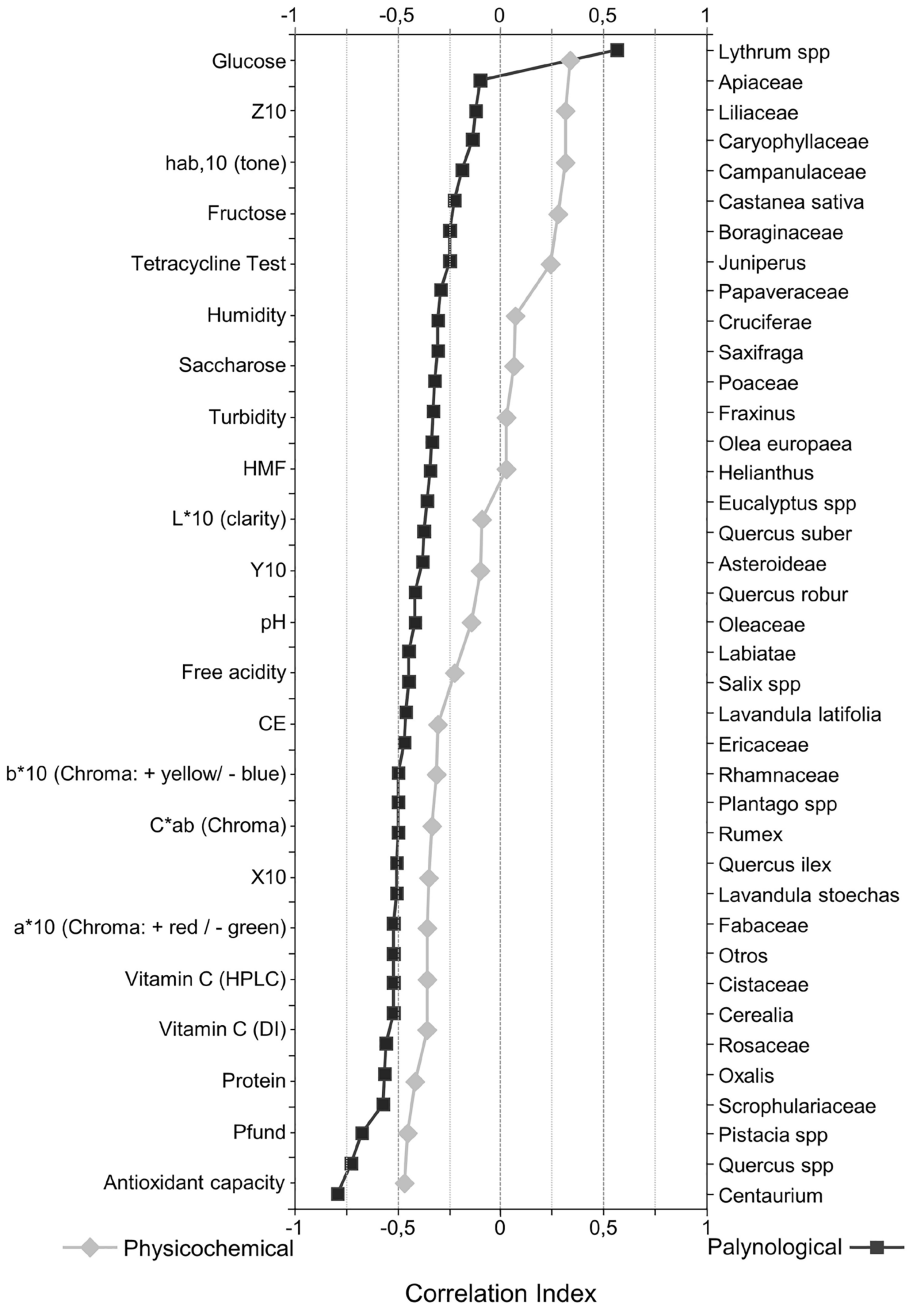
Correlation index: Variable analysis diagram.

After the correlation analyses of the representation of *Echium* pollen type with respect to each of the variables analyzed in the samples, we obtain a first approximation as to which variables will have a stronger influence on the properties of the honey. As a result, there are clearly physicochemical variables that presented correlation indices close to 0, like HMF content and moisture, indicating that these variables have nothing to do with the changes in the proportion of *Echium* pollen in the honey (Fig 2, see also S1 and S2 Tables). The same can be seen with some pollen variables (Apiaceae, Liliaceae,…) that were clearly not related to the relative presence of *Echium* pollen and they were so weakly correlated that they were not considered further to avoid them contributing noise to the analysis. In view of these results, the number of physicochemical variables was reduced from 23 to 9, and the pollen variables from 60 to 22, selecting only those variables that were robustly related to the proportional representation of *Echium* pollen type. Indeed, the objective of this analysis was to summarize the information, eliminating the noise in order to ensure a correct interpretation of the results.

Cohorts were generated in function of the changes in the representation of pollen of the species studied that were in the range of 3%, 5% and 10%. The PCA and Cluster analysis of these cohorts established, with the highest possible precision, the proportion of *Echium* pollen that is most closely related with the physicochemical characteristics studied. After this first filter, the number of samples was reduced based purely on quantitative criteria. Thus, all the samples that did not present information for at least 10% of the variables were excluded, a filter that reduced the number of samples from 157 to 126. Finally, the grouping of the samples into ranges or percentages provided a more comprehensive view of the data, leaving only the problem of expressing the data clearly and concisely.

**Fig 3:**
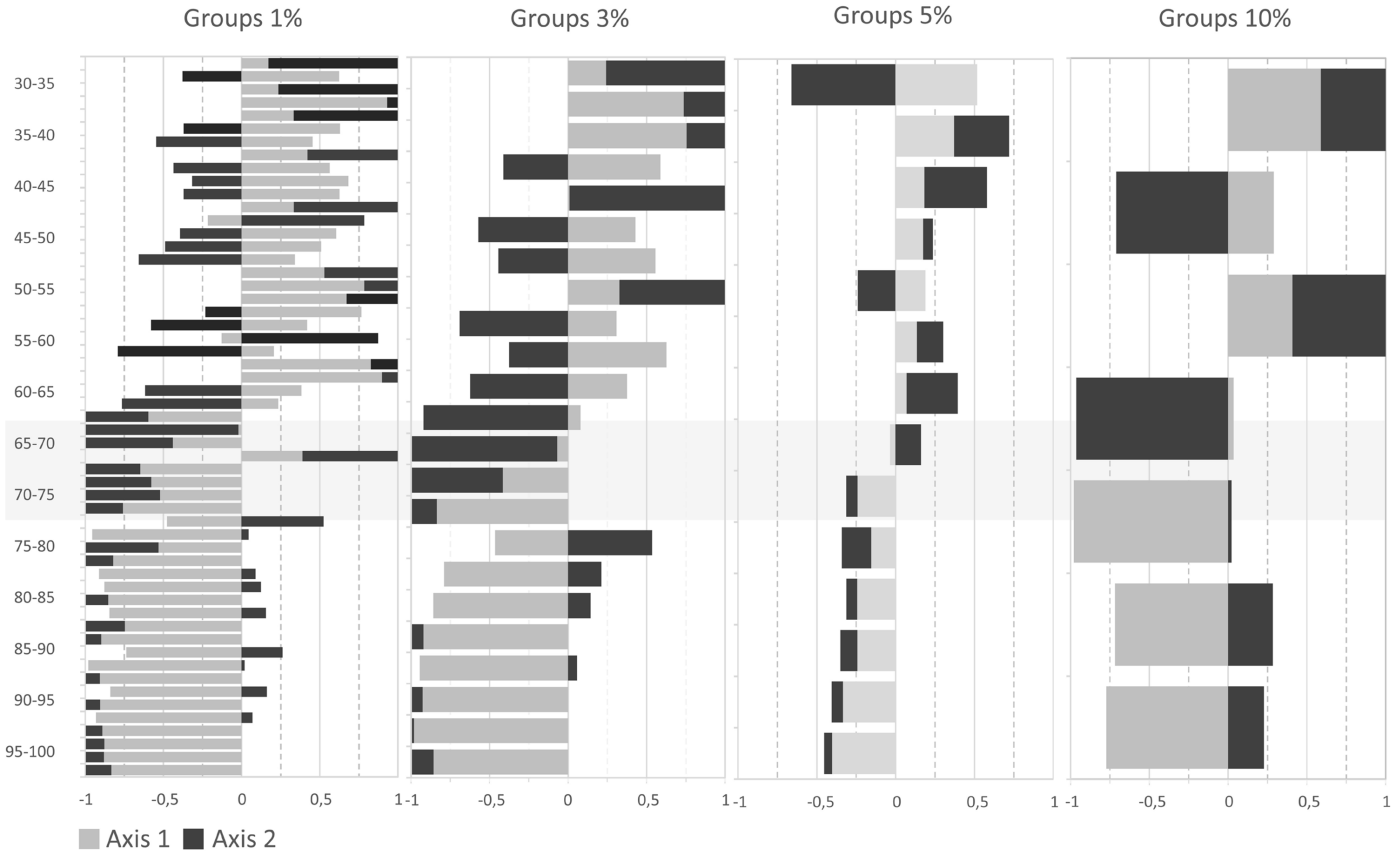
Pollen cohorts.

When the samples were grouped into cohorts that varied by 3%, 5% or 10%, a parallel behavior was evident when the PCA was applied. These analyses were carried out for a maximum of 4 axes and each of the models explains at least 76% of the variance. Ultimately, the cohort of 5% was chosen because it statistically presents a more homogeneous number of samples per segment. A clear distribution of variables was evident in this group, although it did not differ substantially from the distribution in the other groups (Fig 3).

**Fig 4:**
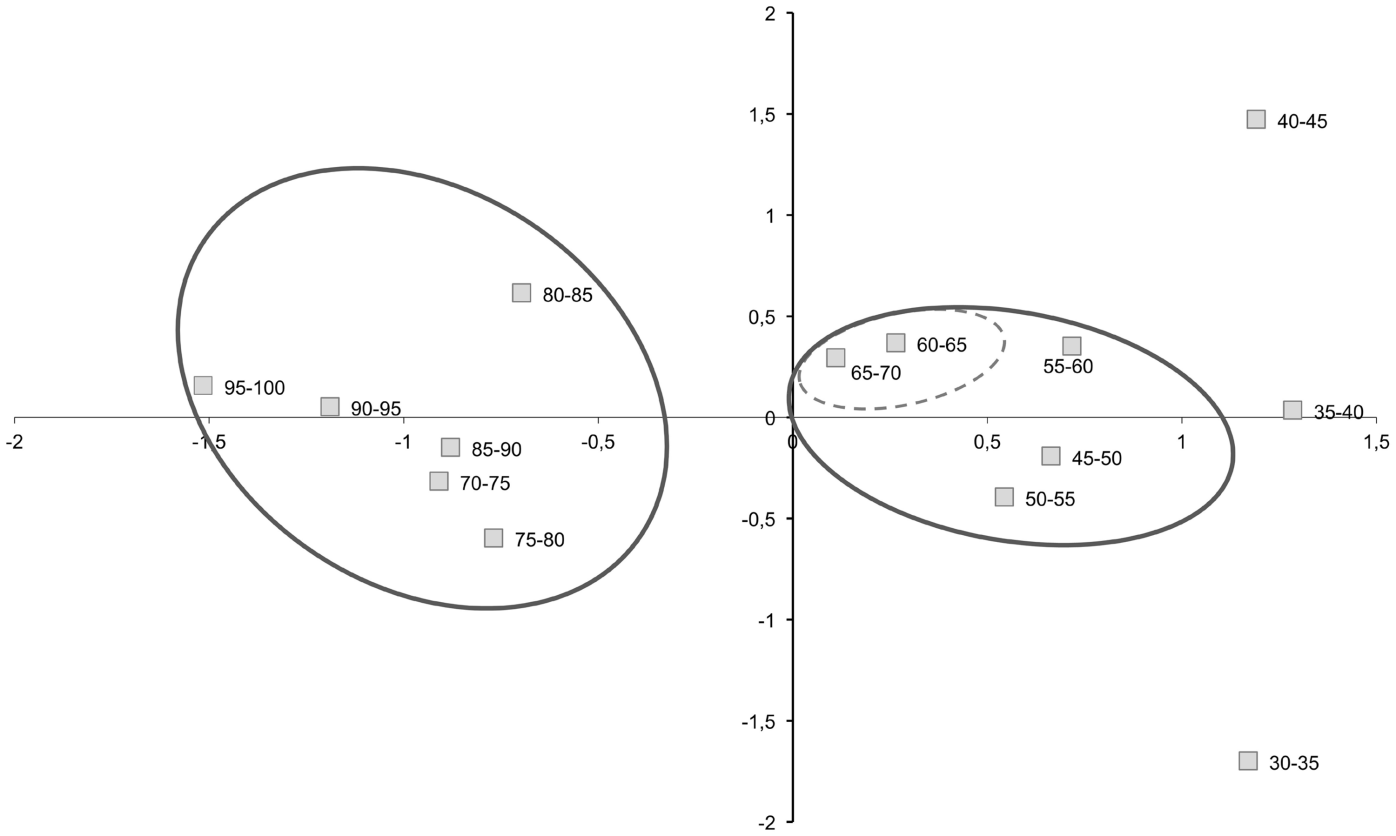
Principal component analysis. Segregation of the honeys in function of the proportion of viper’s bugloss pollen.

In the graphical representation of the PCA (Fig 4), the honeys with a pollen load above 65–70% displayed a distinct behavior in Axis 1. These honeys formed a group with more stable and homogeneous physicochemical characteristics, which distinguished them from the rest of the samples. These samples were situated in quadrants 2 and 3, with negative values of axis 1, while the rest of the honeys had positive values in this axis. In addition, the ranges of 60–65% and 65–70% formed a transitional subgroup, while the ranges with values of 30–35%, 35–40% and 40–45% present an anodyne dynamic.

**Fig 5:**
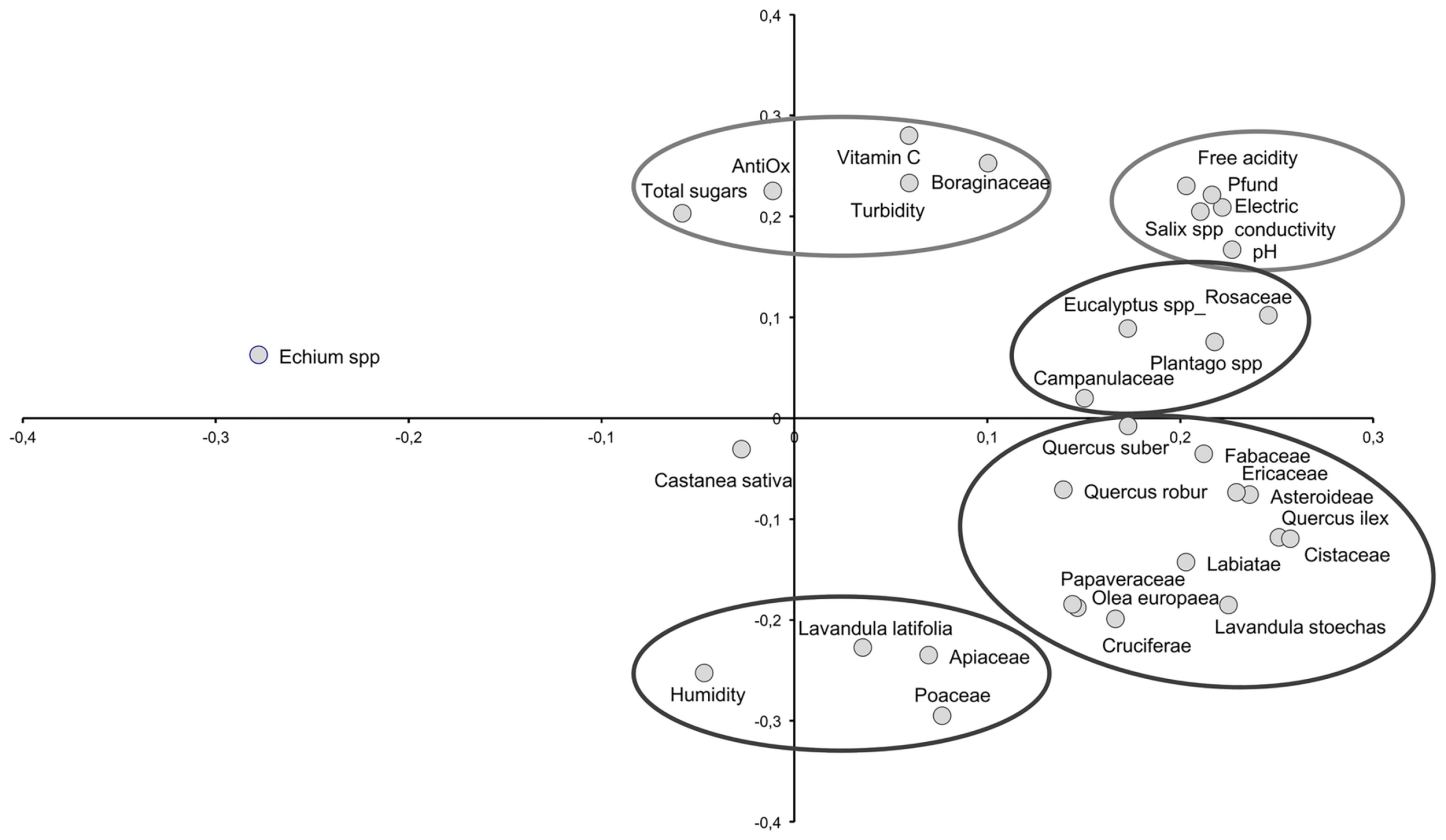
Principal component analysis. Distribution of variables.

This segregation defined the evolution of the honeys with respect to the percentage of *Echium* pollen. We went from samples that had nothing to do with each other (30–45%), to samples with a consistency in terms of their physicochemical and pollinic characteristics (> 70%). A transition phase (60–70%) marked an evolution from multifloral honeys with a high percentages of *Echium* pollen to monofloral *Echium* honeys. As for the distribution, the physicochemical variables were situated in quadrants 1 and 2. This PCA distribution represented 88% of the variance within the first 2 axes (Fig 5).

Variables such as pH, color, conductivity and acidity were negatively correlated with the honeys that had an *Echium* pollen content >70%. By contrast, the turbidity, Vitamin C and sugar content, and the antioxidant capacity were strongly correlated with this type of honey. An increase in the amount of sugars in honey with a high representation of *Echium* has been confirmed previously, with viper's bugloss honeys reported to be richer in fructose and glucose in a study of monofloral honey [51]. This may reflect the local nectar composition, as the nectar of the *Echium* genus, and of the Borraginaceae family in general, is richer in sucrose than that of Rosemary, *Eucalyptus*, *Citrus* and Rosaceae, and as evident in monofloral honeys of these species and in several multifloral honeys [52–54]. Nevertheless, while this Borraginaceae family provides bees with more sucrose, glucose and fructose, the relationship between sucrose and hexoses (fructose + glucose) may be significant [12,13]. In the light of this relationship, the most attractive families are likely to be the Fabaceae, Ranunculaceae and Labiatae (28%, 14% and 4%, respectively) followed by the Borraginaceae, Asteraceae and Dipsacaceae (3%, 2% and 0.5%). The least attractive families in this sense are the Apiaceae (0.2%) and Liliaceae (0.3%: [10]). While the intense production of pollen and nectars by plants in the *Echium* genus does not situate them among the most attractive families, in open habitats, they are still sufficiently attractive as to produce monofloral honeys.

In terms of the antioxidant capacity of the honeys, which given the presence of polyphenols, flavonoids, etc. is not currently questioned [55,17], our data present this activity near the y-axis. All the honeys in our study are spring honeys and hence, their humidity is similar, situating this parameter in the horizontal axis to indicate that there is no relationship or variability. A negative correlation was seen between the pollen content and Pfund values. As the presence of viper’s bugloss pollen increases, honeys become more golden and transparent. This is consistent with the sensory characteristics described previously [17], reflecting a honey with a yellow gold color, and with a light clean taste, a floral bouquet and lemon characteristics.

The pollinic variables were distributed between quadrants 1 and 4, with higher values along axis 1, whereas *Echium* appears at negative values along this axis. Coupled with the segregation of these variables into three groups, this is indicative of a differentiation in function of ecology, plant size and the composition of the vegetation. The herbaceous species and communities rich in *Echium* constitute open subnitrophilous meadowlands (quadrant one), while the decrease in the representation of *Echium* pollen occurs in communities enriched with arboreal or woody species (quadrant four: [2,56,57]).

**Fig 6:**
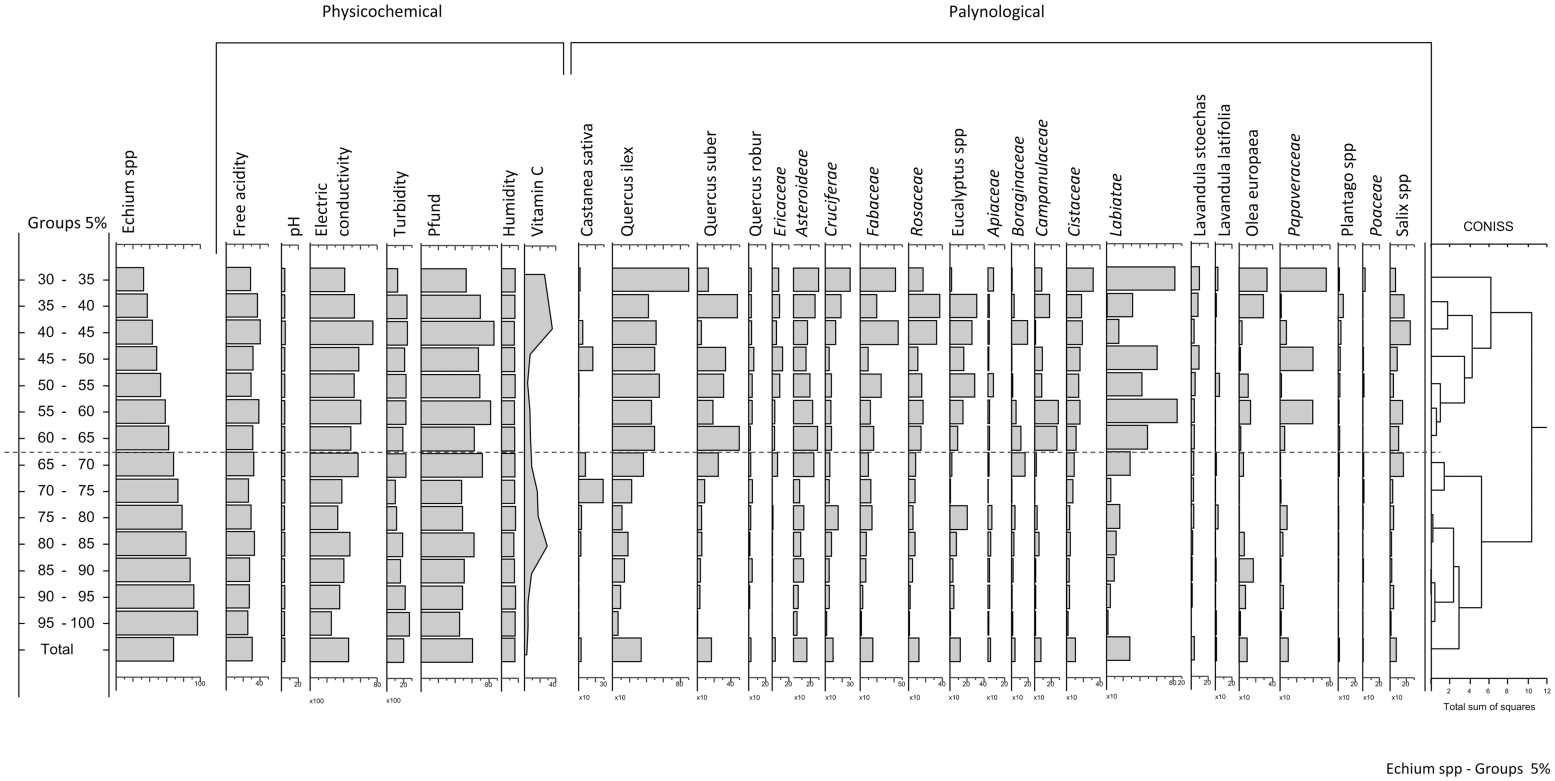
Diagram of the variables: Cluster analysis, Coniss package.

In a diagram of Variables (Fig 6) in which each are serially represented, a new Cluster analysis was carried out using the Coniss package [50]. This analysis segregated the samples into two clearly differentiated groups, where the boundary between the two groups was established at 65% of *Echium* pollen. This corroborates the data obtained previously in the PCA. Analyzing each parameter individually, we can see that the physicochemical parameters vary from 30 to 70% and that they follow nearly parallel Gaussian curves, such that the behavior of these parameters is almost similar to an Echium pollen load of 30% rather than that of 50%. From a representation of 70% Echium pollen onwards, the lines obtained are virtually exponential, indicating a perfect correlation that differs considerably from that seen at 45% representation, the minimum value currently established to consider viper’s bugloss honey monofloral.

## Conclusions

In view of the analysis and results obtained, we found that a representation of Purple Viper's bugloss pollen in honey below 70% can be considered as multifloral. Therefore, we propose that to consider it as monofloral, the percentage of Echium type in the honey sediment should be at least 70%. After a statistical analyses of the physicochemical, sensory and pollen data of 126 honey samples in our study, the parameters that define the monofloral character of Purple Viper's-bugloss honey are essentially: pH, color, electrical conductivity, acidity and *Echium* pollen content. In addition, the monofloral honey of Purple Viper's bugloss produced in the center of the Iberian Peninsula has the following characteristics: high levels of major sugars like hexoses (glucose/fructose) and sucrose; relatively light amber golden honey; and a pollen spectrum rich in herbaceous taxa and low woody pollen taxa.

## Supporting information

S1 Table Means and standard deviations of the groupings of the data of 5%. (PDF)

S2 Table Means and standard deviations of the groupings of the data. (PDF)
