# Supplementary material for: Viper’s bugloss (Echium spp.) honey typing and establishing the pollen threshold for monofloral honey
Source: PLoS One. 2017 Oct 4;12(10):e0185405. doi: 10.1371/journal.pone.0185405 (PMC5627913; doi:10.1371/journal.pone.0185405)
Supplement: S1 Table — (PDF) [file pone.0185405.s001.pdf]

S1 Table. Means and standard deviations of the groupings of the data of 5%

	Echium type	Free acidity	pH	Moisture	Turbidity	electrical conductivity	Pfund
30-35	32,87±1,12	28,72±6,84	4,09±0,35	16,11±1,22	0,13±0,04	0,41±0,2	52,84±20,76
35-40	36,77±1,18	37,06±14,65	4,17±0,22	15,95±0,91	0,24±0,13	0,53±0,25	69,77±26,33
40-45	42,99±1,48	40,15±11,66	4,46±0,13	15±0,58	0,25±0,1	0,75±0,21	85,75±20,52
45-50	48,13±2,01	31,56±8,29	4,26±0,45	15,6±0,18	0,21±0,09	0,58±0,33	67,25±27,34
50-55	52,88±1,37	29,43±5,41	4,25±0,34	16,16±0,99	0,23±0,13	0,53±0,23	69,33±22,81
55-60	58,12±1,54	41,27±13,79	4,32±0,39	15,27±0,81	0,23±0,06	0,71±0,32	83,9±6,93
60-65	62,38±1,48	31,34±11,88	4,13±0,19	15,54±0,76	0,19±0,09	0,49±0,25	62,63±22,49
65-70	68,05±1,23	32,5±7,23	4,22±0,20	15,74±1,34	0,23±0,04	0,57±0,2	72,03±11,16
70-75	73,3±2,21	26,25±12,6	4,04±0,23	15,6±1,00	0,1±0,05	0,38±0,27	47,53±23,51
75-80	77,95±0,74	29,51±6,03	3,84±0,14	16,51±1,32	0,12±0,08	0,33±0,11	48,77±17,23
80-85	82,79±1,41	33,27±10	4,05±0,36	14,85±3,94	0,19±0,08	0,47±0,24	62,17±16,83
85-90	87,74±1,36	24,58±12,56	3,55±1,35	15,51±0,70	0,18±0,1	0,36±0,25	52,03±23,27
90-95	92,04±1,48	27,33±6,88	4,02±0,61	16,31±1,40	0,22±0,12	0,35±0,16	48,84±14,82
95-100	96,15±0,76	23,01±9,29	3,45±1,23	16,19±0,73	0,26±0,16	0,23±0,1	43,84±16,14
