# Supplementary material for: Viper’s bugloss (Echium spp.) honey typing and establishing the pollen threshold for monofloral honey
Source: PLoS One. 2017 Oct 4;12(10):e0185405. doi: 10.1371/journal.pone.0185405 (PMC5627913; doi:10.1371/journal.pone.0185405)
Supplement: S2 Table — (PDF) [file pone.0185405.s002.pdf]

Groupings 3%	Echium	Acidity free	pH	Moisture	Turbidity	Electric conductivity	Pfund
30-33	31,47 ± 0,60	30,58 ± 03,57	4,1 ± 0,45	16,15 ± 2,03	0,13 ± 0,03	0,42 ± 0,18	53,01 ± 12,32
33-36	34,17 ± 0,97	33,68 ± 15,95	4,09 ± 0,31	15,86 ± 0,98	0,19 ± 0,11	0,47 ± 0,29	61,35 ± 30,79
36-39	37,46 ± 0,64	32,85 ± 08,02	4,2 ± 0,18	16,22 ± 0,63	0,2 ± 0,14	0,5 ± 0,17	65,37 ± 20,43
39-42	41,49 ± 0,47	45,43 ± 03,41	4,46 ± 0,14	15,19 ± 0,59	0,26 ± 0,09	0,91 ± 0,09	89,85 ± 04,96
42-45	44,12 ± 0,51	36,19 ± 14,67	4,46 ± 0,15	14,86 ± 0,61	0,24 ± 0,12	0,63 ± 0,21	82,67 ± 28,21
45-48	45,98 ± 0,82	31,63 ± 06,89	4,18 ± 0,42	15,65 ± 0,25	0,23 ± 0,04	0,5 ± 0,39	67,22 ± 36,83
48-51	49,82 ± 0,52	30,57 ± 08,90	4,42 ± 0,50	15,95 ± 0,78	0,21 ± 0,10	0,61 ± 0,3	70,46 ± 24,18
51-54	52,54 ± 0,86	28,09 ± 05,53	4,18 ± 0,36	16,07 ± 1,13	0,21 ± 0,15	0,48 ± 0,26	62,11 ± 24,15
54-57	55,45 ± 1,07	34,97 ± 13,16	4,33 ± 0,37	15,61 ± 0,77	0,26 ± 0,06	0,69 ± 0,35	86,99 ± 06,37
57-60	58,89 ± 1,23	43,70 ± 08,55	4,29 ± 0,28	15,47 ± 0,96	0,24 ± 0,08	0,7 ± 0,12	82,68 ± 04,85
60-63	61,28 ± 0,94	26,02 ± 10,30	4,05 ± 0,21	15,22 ± 0,83	0,17 ± 0,12	0,38 ± 0,23	53,38 ± 22,84
63-66	63,76 ± 0,32	38,00 ± 11,33	4,24 ± 0,11	15,94 ± 0,50	0,22 ± 0,06	0,62 ± 0,24	74,21 ± 18,23
66-69	67,10 ± 0,48	31,50 ± 04,13	4,25 ± 0,15	15,76 ± 1,15	0,23 ± 0,02	0,57 ± 0,11	75,27 ± 03,75
69-72	69,67 ± 0,74	35,38 ± 09,69	4,2 ± 0,25	15,46 ± 1,59	0,2 ± 0,07	0,6 ± 0,27	68,69 ± 14,36
72-75	74,57 ± 0,31	19,38 ± 05,83	3,91 ± 0,01	16,06 ± 0,83	0,09 ± 0,05	0,23 ± 0,06	35,5 ± 15,38
75-78	77,08 ± 0,55	31,35 ± 05,53	3,82 ± 0,13	17,03 ± 1,36	0,09 ± 0,04	0,26 ± 0,10	40,85 ± 15,73
78-81	78,75 ± 0,98	27,57 ± 06,56	3,86 ± 0,14	16,47 ± 1,36	0,15 ± 0,10	0,36 ± 0,09	54,23 ± 16,79
81-84	82,62 ± 1,22	34,13 ± 10,31	3,97 ± 0,30	14,47 ± 4,36	0,18 ± 0,08	0,43 ± 0,18	58,63 ± 16,21
84-87	85,59 ± 0,92	31,15 ± 10,98	4,27 ± 0,43	15,15 ± 0,65	0,19 ± 0,11	0,59 ± 0,36	63,57 ± 32,01
87-90	88,51 ± 0,86	22,38 ± 12,75	3,29 ± 1,62	15,80 ± 0,68	0,18 ± 0,07	0,3 ± 0,21	52,1 ± 17,07
90-93	91,20 ± 0,87	27,31 ± 07,05	4,07 ± 0,71	15,89 ± 1,14	0,18 ± 0,10	0,37 ± 0,18	46,02 ± 16,21
93-96	94,34 ± 0,86	25,03 ± 11,07	3,47 ± 1,33	16,78 ± 1,53	0,26 ± 0,14	0,28 ± 0,11	48,92 ± 12,06
96-99	96,51 ± 0,60	24,16 ± 04,50	3,81 ± 0,20	16,30 ± 0,47	0,31 ± 0,16	0,23 ± 0,07	46,82 ± 18,71

Groupings 5%	Echium	Acidity free	pH	Moisture	Turbidity	Electric conductivity	Pfund
30-35	32,87 ± 1,12	28,72 ± 06,84	4,09 ± 0,35	16,11 ± 1,22	0,13 ± 0,04	0,41 ± 0,20	52,84 ± 20,76
35-40	36,77 ± 1,18	37,06 ± 14,65	4,17 ± 0,22	15,95 ± 0,91	0,24 ± 0,13	0,53 ± 0,25	69,77 ± 26,33
40-45	42,99 ± 1,48	40,15 ± 11,66	4,46 ± 0,13	15 ± 0,58	0,25 ± 0,10	0,75 ± 0,21	85,75 ± 20,52
45-50	48,13 ± 2,01	31,56 ± 08,29	4,26 ± 0,45	15,6 ± 0,18	0,21 ± 0,09	0,58 ± 0,33	67,25 ± 27,34
50-55	52,88 ± 1,37	29,43 ± 05,41	4,25 ± 0,34	16,16 ± 0,99	0,23 ± 0,13	0,53 ± 0,23	69,33 ± 22,81
55-60	58,12 ± 1,54	41,27 ± 13,79	4,32 ± 0,39	15,27 ± 0,81	0,23 ± 0,06	0,71 ± 0,32	83,9 ± 06,93
60-65	62,38 ± 1,48	31,34 ± 11,88	4,13 ± 0,19	15,54 ± 0,76	0,19 ± 0,09	0,49 ± 0,25	62,63 ± 22,49
65-70	68,05 ± 1,23	32,5 ± 07,23	4,22 ± 0,20	15,74 ± 1,34	0,23 ± 0,04	0,57 ± 0,20	72,03 ± 11,16
70-75	73,3 ± 2,21	26,25 ± 12,60	4,04 ± 0,23	15,6 ± 1,00	0,1 ± 0,05	0,38 ± 0,27	47,53 ± 23,51
75-80	77,95 ± 0,74	29,51 ± 06,03	3,84 ± 0,14	16,51 ± 1,32	0,12 ± 0,08	0,33 ± 0,11	48,77 ± 17,23
80-85	82,79 ± 1,41	33,27 ± 10,00	4,05 ± 0,36	14,85 ± 3,94	0,19 ± 0,08	0,47 ± 0,24	62,17 ± 16,83
85-90	87,74 ± 1,36	24,58 ± 12,56	3,55 ± 1,35	15,51 ± 0,70	0,18 ± 0,10	0,36 ± 0,25	52,03 ± 23,27
90-95	92,04 ± 1,48	27,33 ± 06,88	4,02 ± 0,61	16,31 ± 1,40	0,22 ± 0,12	0,35 ± 0,16	48,84 ± 14,82
95-100	96,15 ± 0,76	23,01 ± 09,29	3,45 ± 1,23	16,19 ± 0,73	0,26 ± 0,16	0,23 ± 0,10	43,84 ± 16,14

Groupings 10%	Echium	Acidity free	pH	Moisture	Turbidity	Electric conductivity	Pfund
30-40	34,82 ± 2,30	32,89 ± 11,89	4,13 ± 0,29	16,03 ± 1,04	0,18 ± 0,11	0,47 ± 0,23	61,3 ± 24,59
40-50	45,13 ± 3,11	36,57 ± 10,89	4,38 ± 0,30	15,25 ± 0,54	0,23 ± 0,09	0,68 ± 0,27	78,04 ± 24,33
50-60	54,54 ± 2,85	33,17 ± 10,21	4,27 ± 0,35	15,88 ± 1,01	0,23 ± 0,11	0,58 ± 0,26	73,93 ± 20,21
60-70	64,86 ± 3,19	31,85 ± 09,82	4,17 ± 0,19	15,63 ± 1,02	0,21 ± 0,08	0,52 ± 0,23	66,75 ± 18,52
70-80	76,79 ± 2,39	28,69 ± 07,58	3,89 ± 0,18	16,28 ± 1,27	0,11 ± 0,07	0,34 ± 0,15	48,46 ± 17,79
80-90	84,73 ± 2,82	29,87 ± 11,63	3,85 ± 0,90	15,11 ± 3,08	0,19 ± 0,08	0,43 ± 0,24	58,2 ± 19,75
90-100	93,46 ± 2,36	25,84 ± 07,91	3,82 ± 0,89	16,27 ± 1,20	0,24 ± 0,13	0,31 ± 0,15	47,11 ± 15,20

S2 Table - Means and standard deviations of the groupings of the data
